# Isolation of plant transcription factors using a modified yeast one-hybrid system

**DOI:** 10.1186/1746-4811-2-3

**Published:** 2006-02-22

**Authors:** Sergiy Lopato, Natalia Bazanova, Sarah Morran, Andrew S Milligan, Neil Shirley, Peter Langridge

**Affiliations:** 1 Australian Centre for Plant Functional Genomics, The University of Adelaide, PMB 1, Glen Osmond, SA 5604, Australia

## Abstract

**Background:**

The preparation of expressional cDNA libraries for use in the yeast two-hybrid system is quick and efficient when using the dedicated Clontech™ product, the MATCHMAKER Library Construction and Screening Kit 3. This kit employs SMART technology for the amplification of full-length cDNAs, in combination with cloning using homologous recombination.

Unfortunately, such cDNA libraries prepared directly in yeast can not be used for the efficient recovery of purified plasmids and thus are incompatible with existing yeast one-hybrid systems, which use yeast transformation for the library screen.

**Results:**

Here we propose an adaptation of the yeast one-hybrid system for identification and cloning of transcription factors using a MATCHMAKER cDNA library. The procedure is demonstrated using a cDNA library prepared from the liquid part of the multinucleate coenocyte of wheat endosperm. The method is a modification of a standard one-hybrid screening protocol, utilising a mating step to introduce the library construct and reporter construct into the same cell. Several novel full length transcription factors from the homeodomain, AP2 domain and E2F families of transcription factors were identified and isolated.

**Conclusion:**

In this paper we propose a method to extend the compatibility of MATCHMAKER cDNA libraries from yeast two-hybrid screens to one-hybrid screens. The utility of the new yeast one-hybrid technology is demonstrated by the successful cloning from wheat of full-length cDNAs encoding several transcription factors from three different families.

## Background

Development of plant organs is determined by differential gene expression which can be regulated at many different levels. The major mechanism of differential gene expression is transcriptional regulation [1] which is controlled by transcription factors that bind to DNA *cis*-elements located in gene promoters and/or introns. Analysis of sequenced genomes revealed that transcription factors compose up to 10% of metazoan genomes [2] and more than 5% of the genes in *Arabidopsis *were identified as transcription factors [3]. The use of microarray data and clustering algorithms enables identification of genes with similar expression profiles that may be involved in similar biological processes [4]. Analysis of the promoters of such genes can identify common *cis*-elements, responsible for their co-expression [5]. Nevertheless, these data fail to reveal the transcriptional regulatory mechanisms because of the complexity of the promoter sequences, the relatively small number of *cis*-regulatory elements and transcription factors that have been characterised, and lack of data about cooperative regulation of gene expression in plants by several transcription factors simultaneously. To understand transcriptional mechanisms that lead to differential gene expression, genes have to be experimentally coordinated with the corresponding TFs that regulate their expression.

A powerful method of identifying protein-DNA interactions is the yeast one-hybrid (Y1H) system which is a variant of the yeast two-hybrid system (Y2H) [6-8] Like the Y2H, the Y1H is based on the domain structure of eukaryotic transcription factors, consisting of a transcription activation domain (AD) and a DNA binding domain (BD). A part of the promoter or tandem copies of putative transcription regulatory DNA elements are cloned upstream of a reporter gene in a reporter vector. In the latest version of the method, part of the reporter vector containing the *cis*-element of interest upstream of the reporter gene is introduced into the yeast genomic DNA *via *homologous recombination with a non-essential gene [9]. This stabilises the results of the Y1H screen and brings the protein-DNA interaction into the natural environment of the nucleus. The second component of the Y1H system is shared with the Y2H system. This is a cDNA library which expresses a fusion protein of a constitutive activation domain and a variable DNA binding domain encoded by the appropriate cDNA. The Y1H reporter strain is transformed with a cDNA library vector, and interaction between target DNA and hybrid protein is detected by reporter gene expression [10].

The advantage of the Y1H screen compared to biochemical *in vitro *techniques, such as DNA affinity chromatography, or the identification of DNA binding proteins seen in electrophoretic mobility shift assays using high-resolution two-dimensional electrophoresis coupled with MS [11], is that the procedure does not require specific optimisation of *in vitro *conditions. This method allows examination of protein-DNA interactions *in vivo *in the conditions inside the nucleus of the eukaryotic cell. This environment often provides correct folding and modifications of the prey protein [12]. The Y1H system also enables the use of known *cis*-elements as well as non-characterised fragments of promoters to search for specifically interacting DNA-binding proteins in expressional cDNA libraries [10,13]. DNA-binding proteins that do not function in transcription and lack a native AD (such as chromatin remodelling, replication and DNA repair proteins, and enzymes involved in transposable elements movement) can also be found using this approach, as a strong, heterologous AD is added to the prey protein [14,15]. This method enables the cloning of families of factors with conserved DNA binding domains which can interact with the same *cis*-activation DNA element [16-19]. The screening of several millions of colonies simultaneously makes the Y1H system extremely sensitive, allowing the cloning of very low abundance transcription factors which may be absent from even very large EST databases.

To clone a group of specific factors one can make the library from a particular tissue, part of a tissue or a group of cells. Methods for the preparation of such libraries, from limited amounts of starting material, were developed recently [8,20] and kits for their preparation are available commercially. One such kit, the MATCHMAKER Library Construction Kit 3 (Clontech™, Palo Alta, CA), provides a simple method for the construction of cDNA expression libraries for Y2H screening. The library construction protocol integrates the efficiency of SMART cDNA synthesis technology [8,20,21] with the simplicity of cloning using homologous recombination in yeast [22].

Although the MATCHMAKER Library Construction Kit provides a facile method for Y2H screening, the application of pre-made MATCHMAKER cDNA libraries to the Y1H method has not been proposed. Here we describe an adaptation of a straightforward and efficient version of the Y1H method [9], enabling the screening of MATCHMAKER cDNA libraries for protein/DNA interactions. The cDNA library used in the Y1H screen was prepared from a small amount of total RNA isolated from the liquid part of wheat endosperm before and during cellularisation. We demonstrate the efficiency of the method by cloning several wheat transcription factors using three different tandem repeats of a known DNA *cis*-element as bait.

## Results

### Adaptation of the Y1H system to screen expressional cDNA libraries prepared in yeast

To facilitate the cloning and characterisation of transcription factors from wheat, a plant with a very large and unsequenced genome, we combined one of the existing Y1H systems with a quick and relatively simple method of preparing cDNA expression libraries

The cDNA library was prepared from 2 μg of total RNA isolated from the liquid part of the wheat endosperm coenocyte collected before and during cellularisation, at 3 to 7 days after pollination (DAP), and was originally designed for the two-hybrid screen based upon yeast mating. It was prepared using the Yeast MATCHMAKER System 3, which enabled the quick and relatively easy construction of representative libraries from a small amount of tissue by homologous recombination-mediated cloning in yeast.

**Figure 1:**
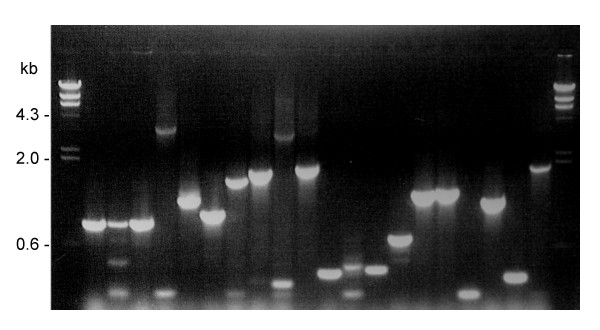
Size of inserts from 24 randomly picked colonies of the expressional library prepared from the wheat syncytial endosperm.

As the first libraries prepared according to the Clontech™ manual contained too many inserts smaller than 600 bp, we introduced a small change into the method and used CHROMA SPIN+TE-1000 (Clontech™) instead of CHROMA SPIN+TE-400 columns for cDNA size fractionation. According to the CHROMA SPIN Columns User Manual (PT1300-1), a single purification on the TE-1000 column removes about 60% of cDNA smaller than 1 kb. This change in the protocol substantially increased the the average length of cDNAs and thus the frequency of full-length cDNAs in the library in comparison with the original method, while still preserving good representation of short cDNAs. Results of the PCR analysis of the length of cDNA inserts from 24 randomly selected library clones are shown in Fig. 1. The library was prepared without amplification and stored as aliquots of the yeast glycerol culture. It has been used for Y2H and Y1H screens for more than 4 years after preparation without any noticeable changes in quality.

Y1H reporter strains were prepared by cloning three to four tandem repeats of: 1) the *cis*-elements specific for two classes of homeodomain transcription factors (HDZipI-II) [23,24]; 2) the Drought Responsive Element (DRE) from the promoter of the *rd29A *gene from *Arabidopsis thaliana*, specific for the DREB/CBF subfamily of single AP2-domain proteins [25]; and 3) the E2F binding *cis*-element from the promoter of the ribonucleotide reductase (*RNR*) gene of tobacco [26].

The use of repeats of *cis*-elements helps to optimise the distance between the plant regulatory element and the minimal promoter of the yeast GAL4 gene.

Oligonucleotides with complementary sequences for each tandem of *cis*-elements (Table 1) were designed so that after annealing they had protruding ends which were used for cloning into *Not*I-*Spe*I sites of the pINT1-HIS3NB binary vector (unpublished, kindly provided by Dr. PBF Ouwerkerk). The pINT1-HIS3NB vector was obtained by cloning the *Not*I-*Bam*HI fragment of the pHIS3NB cloning vector into the *Not*I-*Bcl*I sites of the pINT1 integration vector [9,12]. This enabled single step cloning of *cis*-elements into the unique *NotI *and *Spe*I restriction sites of the binary vector. Constructs containing inserts were selected by PCR using primers from the vector. Empty vector served as a template for the control of fragment size.

**Table 1:** Oligonucleotides used for the generation of bait sequences. Tandem repeats of *cis*-elements are shown in bold, with a single element underlined.

***cis*-element repeat**	**Forward primer** ** sequence**	**Reverse primer** ** sequence**	**References**
HD-Zip class I and II	GGCCGC**CAATAATTGCAATGAT- TGCAATTATTGCAATCATTG**A	CTAGT**CAATGATTGCAATAAT-** **TGCAATCATTGCAATTATTG**GC	23, 24
DRE, AP2 domain proteins	GGCCGC**TACCGACATTACCGAC-ATTACCGACATTACCGACAT**A	CTAGT**ATGTCGGTAATGTCGG-TAATGTCGGTAATGTCGGTA**GC	25
OsE2Fa	GGCCGC**TTTTCCCGCCTTTTTT-CCCGCCTTTTTTCCCGCCTT**A	CTAGT**AAGGCGGGAAAAAAGG-CGGGAAAAAAGGCGGGAAAA**GC	26

In order to simplify electrophoretic analysis of the small sized inserts, PCR primers were designed to anneal approximately 50 bp either side of the cloning sites, giving an amplicon from empty vector of about 100 bp. Plasmids with inserts were subsequently analysed by sequencing using the same primers. Integration of the construct into the yeast genomic DNA was performed as described in [12]. Reporter strains, designated yHDZipI-II, yDRE, and yE2F, respectively, were incubated overnight in rich media, and stored as 1 ml aliquots of glycerol culture at -80°C until use. Before screening, a yeast glycerol culture was thawed at room temperature and incubated for several hours in 50 ml YPDA media at 30°C, with shaking, to revive yeast cells. This increased the efficiency of mating the reporter strain with the strain containing the expressional library.

**Figure 2:**
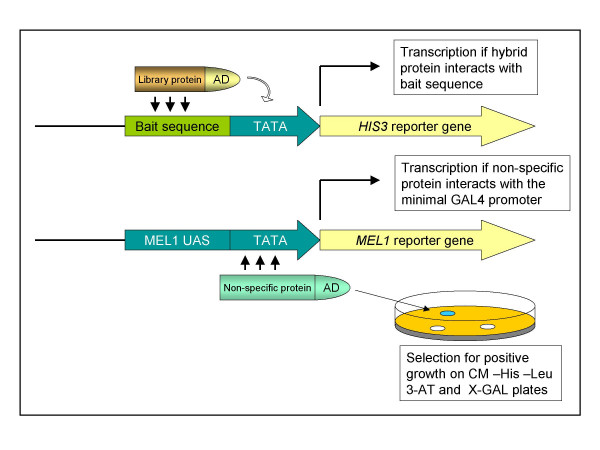
Diagrammatic representation of the basis for the MEL1 selection strategy.

cDNA expression libraries for the Y1H screen were usually made in *E. coli *using binary vectors which are able to replicate in both bacteria and yeast. To make a screen, pools of library plasmid DNA purified from bacteria were transformed into the yeast reporter strain. Unfortunately, cDNA libraries prepared directly in yeast cannot be used for the isolation of large amounts of the library plasmid DNA, and thus the Y1H screen can not be performed using plasmid transformation. To perform the Y1H screen, we mated each bait yeast reporter strain, based on Y187 (*MATa*), with the Yeast MATCHMAKER System 3 cDNA library prepared in the yeast strain AH109 (*MATα*). This approach provided us with the capacity to use, in yeast one-hybrid screens, cDNA libraries prepared according to the method proposed by Clontech™. In addition, mating with the AH109 strain enabled us to omit the labourious β-galactosidase filter assay. Rather, we used *MEL1 *[27], one of the reporter genes of this strain, together with direct addition of the α-galactosidase substrate X-α-GAL into selective media, to stain false positives (blue colonies) on plates during the second round of selection for positive clones. The promoter of *MEL1 *contains no plant *cis*-elements and does not interact specifically with transcription factors, but does interact with a large group of false positives, which interact with the MEL1 minimal promoter (Fig. 2).

**Figure 3:**
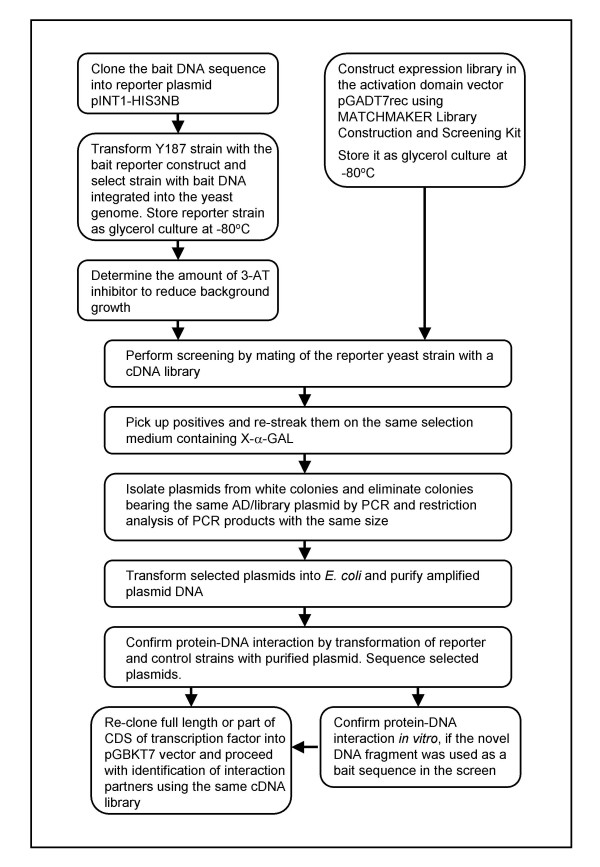
Flow diagram showing the strategy for the Y1H screen and analysis of positive clones.

An additional advantage of this method is that the same cDNA library, prepared from endosperm, was subsequently used for the further characterisation of the isolated transcription factor genes. After re-cloning selected transcription factor cDNAs into the pGBKT7 vector (Clontech™), they were used as bait for the identification of their interacting partners *via *the Y2H screen (data not shown). The strategy of the Y1H screen as well as selection and analysis of positive clones is shown in Fig. 3.

### Results of the Y1H screens

To test the method, we chose specific *cis*-regulatory elements for families of transcription factors which are of interest in our laboratory. These are factors known to be involved in the regulation of cell fate (HDZip TF), cell proliferation and cell size (E2F), and factors which can potentially be involved in the protection of the developing grain from drought or cold stress (DREB/CBF TF). Some of these *cis*-elements were successfully used as a bait in Y1H screens to clone HDZip and DREB factors from other plant species [13,19,28,29]. For others, such as E2Fs, specific interaction between *cis*-elements and transcription factors has only been demonstrated *in vitro*, using gel shift assays [26,30,31]. The list of *cis*-elements used in this paper is shown in Table 1.

**Figure 4:**
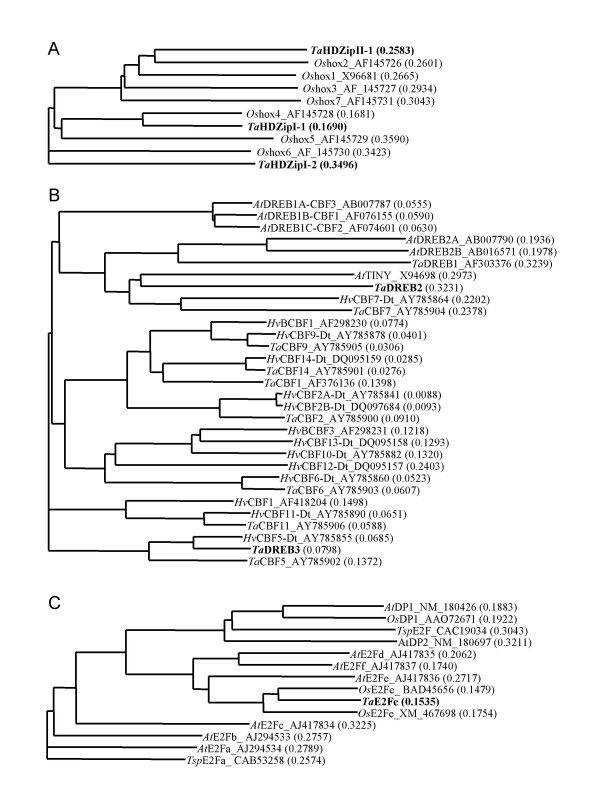
**Diagrammatic representation of sequence similarity between proteins identified in the Y1H screen and selected, known members of each family from different plants. **A – HDZip, B – DREB, and C – E2F. Accession No of cloned cDNAs: *Ta*DREB2 [GenBank DQ353852], *Ta*DREB3 [GenBank DQ353853], *Ta*E2Fe [GenBank DQ353854], TaHDZipI-1 [GenBank DQ353855], TaHDZipI-2 [GenBank DQ353856], TaHDZipII-1 [GenBank DQ353857], TaMYB1 [GenBank DQ353858].

More than 100 positive clones were isolated in the screen with the yHD-ZipI-II bait strain. The clones encoded full and partial length cDNAs of three different proteins. Two were designated *Ta*HDZipI-1 and *Ta*HDZipI-2 and represented 20% and 76% of all clones, respectively. Sequence analyses of the longest clones showed the cDNAs to be 1.0 and 1.1 kb, encoding proteins of 267 and 333 amino acids, respectively. Comparative sequence alignments with seven HDZip (*Os*hox) proteins from rice [13,32] were used to confirm that the full-length open reading frames of each gene had been cloned. These alignments revealed *Ta*HDZipI-1 and *Ta*HDZipI-2 to be most similar to rice *Os*hox4 and *Os*hox6, respectively, and identified *Ta*HDZipI-1 and *Ta*HDZipI-2 as homeodomain-leucine zipper subfamily I proteins (Fig. 4A).

The remaining clones were represented by four independent clones (4%) containing 1.2 kb inserts encoding the full length coding region (279 amino acids) of a class II HDZip factor. This was designated *Ta*HDZipII-1. It has the highest level of protein sequence identity with *Os*hox2 from rice.

**Figure 5:**
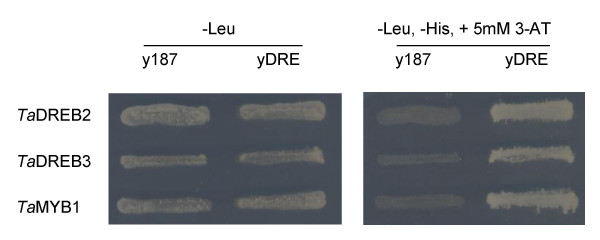
Example of confirmation of protein -DNA interaction for MYB and DREB proteins by the Y1H assay.

About 25 colonies were isolated in the Y1H screen using repeated DRE as bait. cDNAs (with full length coding regions) of three transcription factors were isolated from white colonies after a second round of selection. Two novel DREB factors, designated *Ta*DREB2 and *Ta*DREB3 were cloned from the cDNA library prepared from the liquid part of the syncytial endosperm of wheat. The first was either an orthologue or close homologue (38.8 % identity in amino acid sequences) of TINY from *Arabidopsis*. This is a single AP2 domain factor involved in plant development [33]. *Ta*DREB3 is a novel single AP2 domain transcription factor with 77.7% amino acid sequence identity to the *Hv*CBF5 [34]. The relationship between these sequences is shown in Fig. 4B. Interestingly, the third transcription factor isolated in this screen was a putative Myb transcription factor, designated *Ta*Myb1. The 1182 bp insert encodes a 298 amino acid protein which belongs to the R2R3 group of Myb factors. Two independent clones encoding the same *Ta*MYB were found in the screen. As MYB factors have not previously been associated with the DRE element, we decided to conduct more tests to confirm that this protein indeed interacts with the drought responsive element. To demonstrate this, Y187, AH109 and each of the three yeast strains described in this paper, with different plant *cis*-elements, were transformed using the plasmid containing *Ta*MYB cDNA; we found that the expression of *Ta*MYB supports growth on selective media of the yDRE strain only, and does not support the growth of the other four strains. The interaction of isolated DREB and MYB factors with DRE element in the Y1H system is shown in Fig. 5.

Several hundred colonies were selected in the Y1H screen with the E2F *cis*-element as bait (yE2F strain). Ninety-six colonies were used for the second round of selection and 94 were white on media with X-GAL. The cDNA inserts of white colonies were analysed by PCR and restriction using *Hae*III to show that all 94 colonies contained plasmids with the same insert. The insert encoded an E2F transcription factor with the highest level of amino acid identity to E2Fe (DEL1) from *Arabidopsis*. This is an important inhibitor of the endocycle, which preserves the mitotic state of proliferating cells by suppressing transcription of genes that are required for cells to enter the DNA endoreduplication cycle [35,36]. All clones contained the full length coding region; the length of the isolated cDNA is 1508 bp and encodes a 423 amino acid protein, designated *Ta*E2Fe. Alignment of the amino acid sequence of *Ta*E2Fe to E2Fe factor from *Arabidopsis *is shown in Fig. 4C.

### Q-PCR analysis of expression levels of identified proteins

The spatial and temporal expression patterns of isolated wheat homeobox-containing proteins and DREB proteins were determined by real-time or quantitative PCR (Q-PCR). cDNAs used for these analyses were derived from a variety of vegetative and floral tissues, grain at different stages of development (1–20 DAP), and fractions of the grain at 5 DAP enriched either for the embryo, the liquid part of the syncytial endosperm or the outer layers of the grain (testa, pericarp, *etc*.).

The data demonstrated a correlation between the abundance of mRNAs of cloned transcription factors in the plant tissue sample and the number of independent clones of the respective cDNAs obtained from the cDNA library prepared from this tissue.

#### The HDZip family of genes

**Figure 6:**
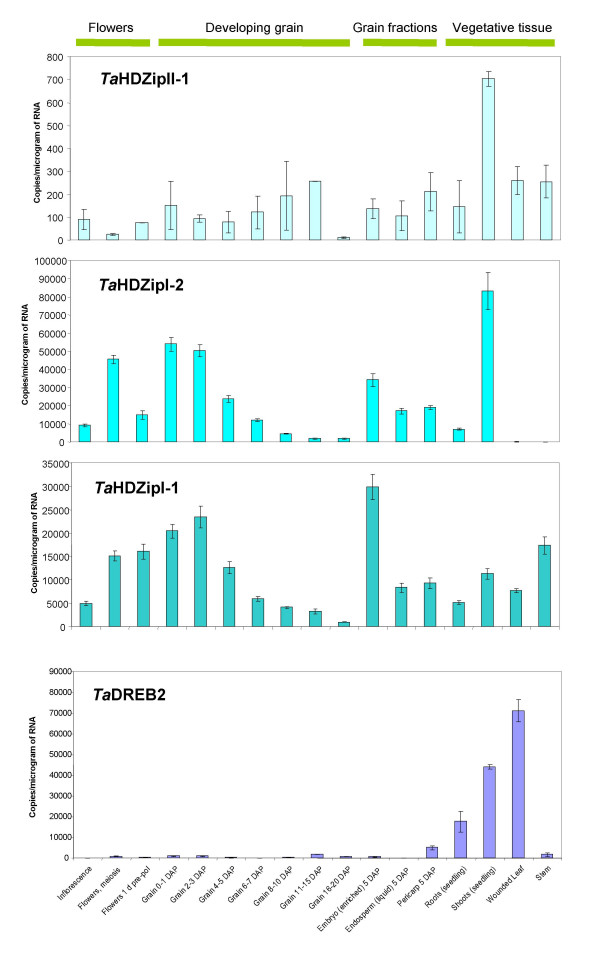
Expression of *HDZip *and *DREB *transcription factors at different stages of flower and grain development, in grain fractions and vegetative tissue, as determined by Q-PCR

*Ta*HDZipI-1 and *Ta*HDZipI-2 showed a similar expression profile in developing flowers, grain, and grain fractions, except *Ta*HDZipI-1 displayed stronger expression in the embryo enriched fraction relative to the liquid part of the endosperm (Fig. 6). However, expression levels of these proteins in other plant tissues differed considerably. *Ta*HDZipI-2 was strongly expressed in shoots of 10 cm long seedlings, and was absent in mature tissues. In contrast, *Ta*HDZipI-1 was expressed in both seedlings and mature tissues with maximal expression in the stem. The *TaHDZipI-1 *gene was most highly expressed in the embryo enriched fraction whilst *TaHDZipI-2 *was expressed predominantly in seedling shoots and in the first three days of grain development. The transcript abundance of *TaHDZipII-1 *was found to be very low (bordering the limits of detection for the method) in all tissues with the highest level of expression in seedling shoots.

Abundance of mRNAs of *Ta*HDZipI-1, *Ta*HDZipI-2 and TaHDZipII-1 in the liquid fraction of endosperm isolated at the 5 DAP is about 18000, 8000 and 100 copies per μg of total RNA, respectively. This correlates well with the abundance of their clones isolated in the Y1H screen from the cDNA library.

#### The DREB family of genes

mRNAs of both *Ta*DREB2 and *Ta*DREB3 have a very low level of abundance in all examined tissues of unstressed wheat (several copies per μg of total RNA according to the Q-PCR data) and are therefore expected to be represented by a relatively low number of plasmids in the cDNA library. This correlates with our results (Fig. 6). The results of Q-PCR for *Ta*DREB3 are not shown in the figure as the data were well below the limit of detection for the method. Nevertheless, the cDNA of *Ta*DREB3 was cloned because the screening of a high number of clones (about six million) facilitates the isolation of even very low abundance cDNAs.

The expression pattern of *Ta*E2Fe has not been studied yet.

## Discussion

### Method improvement

Since the development of the reverse transcription and polymerase chain reaction (RT-PCR) and genomic and EST databases for several plants, isolation of target genes has become a routine procedure. Unfortunately, one of the problems is that the application of RT-PCR is limited to plants for which extensive EST databases exist and where mRNAs from genes of interest are sufficiently abundant to be represented in databases by several ESTs. This is not always the case with rare transcripts such as those for many transcription factors. Another problem which arises in work with plants containing large or polyploid genomes, such as bread wheat, is isolation of full length sequences of long cDNAs. Application of both RACE-PCR and Genome Walking is labour intensive and is often not very efficient. Alternative methods of cDNA cloning are based on the utilisation of properties of DNA and proteins that specifically recognise and bind other molecules through the screening of cDNA expression libraries. Among these methods is the yeast one-hybrid system which *inter alia *can be used for the isolation of novel genes, which encode proteins that bind to a DNA target sequence such as *cis*-regulatory elements.

The Y1H assay is highly sensitive because detection of the interactions between the protein and DNA target occur while proteins are in their native configurations *in vivo*. The most evolutionally conserved *cis*-elements characterised across several plant species (for example, *Arabidopsis*, rice or maize) can be used for isolation of orthologues and homologues from phylogenetically distant plants as well as plants with complex or multiple genomes, especially when PCR methods are not efficient because of the absence of *in silico *data.

In this paper, we successfully used DRE from *Arabidopsis *and E2F *cis*-element from tobacco to isolate wheat transcription factors. Since this method allows the screening of several millions of colonies simultaneously, the Y1H system allows the cloning of full length cDNAs of DNA binding proteins which have very low levels of expression. Based on these advantages, the Y1H method was chosen to clone full length transcription factors from the developing endosperm of wheat.

To get the maximum benefit from this method, the original Y1H protocol was modified. The first improvement was introduced in the method of screening. The Y1H is typically labour intensive as it relies on the isolation of the library plasmids from the bacterial library host strain followed by transformation of the isolated library plasmids into the reporter yeast strain to allow the protein-DNA interaction. Although lambda-based *E. coli*-yeast shuttle vector libraries do have their advantages, for example the possibility to introduce the library into different yeast strains, there are several disadvantages: 1) the methods of library preparation in bacteria are usually more labourious than directly in yeast; 2) the need to amplify libraries for the isolation of large amounts of plasmids, which reduces the quality of the libraries; and 3) the labourious and not always efficient large scale yeast transformation must be repeated for each screen.

The modification of the protocol for Y1H screening presented here involves utilising a yeast mating step to introduce the library plasmid and reporter construct into the same cell. This modification improves the efficiency of the screen, prevents unnecessary amplification of the cDNA library and eliminates the labour intensive steps of phage infections, growing *E. coli *cultures and making plasmid maxipreparations. A representative cDNA library enriched for full length cDNAs can be prepared from a few μg of total RNA directly in the yeast strain (*MATa*) as proposed in the Clontech™ MATCHMAKER manual. The library is then screened by mating with a reporter strain (*MATα*). Use of the library without amplification leads, as demonstrated in this paper, to a correlation between the abundance of particular mRNAs in the original RNA and in the library prepared from this sample.

A second modification of the method was introduced in the cDNA library preparation. Utilisation of cDNA size fractionation columns other than those suggested in the Clontech™ protocol substantially enriched the library with full length sequences. All cDNAs reported here were represented by both partial and full length clones. Only one cDNA from 15 cDNAs of transcription factors cloned in our laboratory from such libraries had a partial open reading frame. The longest full length cDNA was 3.2 kb long (data not shown).

The third improvement comes from the utilisation of AH109 strain, which contains the *MEL1 *reporter gene under its own promoter containing a Gal4p binding site. The *MEL1 *gene encodes α-galactosidase, which in contrast to β-galactosidase is secreted from the yeast cell and its activity can be easily detected by direct addition of substrate to selective media instead of the labour intensive filter-lift assay for β-galactosidase. The *MEL1 *gene was successfully used as a negative reporter to eliminate the group of proteins interacting with the minimal promoter of GAL4, which is situated in the bait construct downstream of the *cis*-element of interest. However, in some rare cases, plant transcription factors can weakly activate the GAL4 promoter and colonies of interest can become slightly blue after several days of growth on the media with substrate. We recommend including such colonies in further characterisation of cloned sequences.

### Results of Y1H screen

Repeats of the nine base pair sequences 5'-CAATNATTG-3' with either A/T for HDZip class I or G/C for HDZip class II in the middle position [23,24] were successfully used earlier in a Y1H screen of a cDNA library prepared from rice embryos at 6 DAP [13]. Although, we have found that both class I and class II HDZip transcription factors recognise each of four sequences in the Y1H screen (data not shown), baits used in this paper contained four repeated *cis*-elements, each time with a different nucleotide in the middle position. Abundance of the clone for each particular isolated HDZip factor in the cDNA library was correlated with the abundance of mRNA in the tissue used for library preparation. This confirms the good quality of the library.

Because of the relatively high abundance of *Ta*HDZipI-1 and *Ta*HDZipI-2 in the library and the strong interaction of HDZip proteins with the specific *cis*-element, the occurrence of false positives was very low. Unfortunately, because of the relatively abundant sequences, tens or even hundreds of clones have to be analysed to find extremely rare cDNAs. For example, only three *Ta*HDZipII-1 clones were found in around 100 analysed colonies. Neither use of other baits with slightly changed sequences nor PCR analysis of more clones revealed other HDZip factors of class I and II in wheat syncytial and cellularising endosperm at 3 to 7 DAP (data not shown).

Several AP2 domain containing transcription factors from the DREB/CBF subfamily were isolated from *Arabidopsis *[28,29] and maize [19] with the help of theY1H system. The triple DRE *cis*-element from *Arabidopsis *has been successfully used in the Y1H system to confirm the interaction of *Ta*DREB1 with the Drought Responsive Element from *Arabidopsis *[37]. Here we demonstrate successful isolation of two DREB proteins from wheat endosperm using the same *Arabidopsis cis*-element repeat 5'-TACCGACAT-3'. This implies that the DRE element can be used to obtain cDNAs of DREB proteins from various plant species, as the DRE motif itself is highly conserved in all plants studied [38].

Although DREB proteins are induced by different environmental stresses, very low levels of their mRNAs can often be found in unstressed tissues [39]. This can be explained either by the involvement of some of these transcription factors in plant development, or the naturally low amounts of DREBs can be important for the positive regulation of their own expression. It may also be that the low amount of some DREB factors is important for negative regulation of the expression of other DREB proteins during stress [40]. It was therefore not surprising that one of the isolated factors is either an orthologue or close homologue of *TINY *from *Arabidopsis*, a DREB factor which is involved in developmental processes. An *Arabidopsis *mutant overexpressing TINY demonstrated decreased cell size resulting in the "tiny" size of the whole plant [33]. Another factor isolated in the screen, *Ta*DREB3, has some level of identity to cold induced CBF factors, although its function is still unknown. Currently, both factors are being tested for their induction in response to environmental stresses.

The small number of clones obtained in the screen with DRE correlates with the extremely low amount of mRNAs encoding DREB2 factor in the unstressed syncytial endosperm as detected by Q-PCR (Fig. 6). The abundance of DREB3 in the cDNA is below the sensitivity of the Q-PCR method in all tissues analysed and is not shown here. Low abundance of DREB sequences in the cDNA library led to smaller numbers of positive clones and higher numbers of blue false positives, the main types of which will be discussed below.

Surprisingly, using DRE element as bait, we also isolated several independent clones of the MYB transcription factor designated *Ta*MYB1. Using the Y1H assay and different reporter strains it was confirmed that *Ta*MYB1 interacts with either DRE or part of DRE. Several MYB-like proteins were reported to be isolated from plants using a Y1H screen [41-43]. However, none of the *cis*-elements that were used as bait has a high level of identity to DRE.

*Ta*MYB1 belongs to the R2R3 group of MYB factors as it contains two DNA-binding domains in the N-terminal region of the protein. The R2R3 group is the largest in plants with over 100 estimated TFs in *Arabidopsis *[44]. Members of this group were found to be involved in the regulation of secondary metabolism, plant morphogenesis, signal transduction in plant growth, abiotic stress and pathogen defense [45]. Different target recognition sites were identified for different groups of MYB proteins as well as within the same group [45]. The sequences of most MYB binding sites are different from that of DRE. However, MYB proteins are known to have considerable inherent flexibility in their ability to recognise target sites [46].

The DRE is present in many abiotic stress response genes and it is conceivable that some members of the MYB family of transcription factors regulate the coordinated response of plants to drought stress through the same element. A MYB factor *At*MYB2 has been demonstrated to be involved in the regulation of the drought and ABA stress response in *Arabidopsis *[47]. *At*MYB2 is induced by drought and also during seed maturation and interacts *in vitro *with DNA elements containing a consensus MYB recognition sequence 5'-TAACTG-3'. Although it belongs to the same subfamily of Myb proteins as *Ta*MYB, it is not a close homologue of *Ta*MYB (23.8% amino acid sequence identity). It is also possible that *Ta*MYB is not a stress inducible protein but involved in the regulation of developmental processes in the absence of stress through the DRE or part of the DRE of several genes. It might be exchanged for more abundant or more specific DREB factors as soon as the plant is exposed to stress. Although we have not yet identified the specific DNA sequence which interacts with *Ta*MYB, we do not believe that it is the core sequence of the DRE, but probably the sequence generated between the borders of DRE repeats.

The *cis*-element for E2F proteins is highly conserved in all organisms. It was reported that the canonical *cis*-element from human promoters interacts with plant E2F factors *in vitro *[48]. E2F factors from *Arabidopsis *interact *in vitro *with the *cis*-element from a tobacco promoter [30]. The Y1H system has not been used previously for isolation of E2F cDNAs. However, it was used to demonstrate interaction of human E2F-1 with the WDV2 *cis*-element from the large intergenic region of the wheat dwarf virus (WDV) [49]. We could not isolate any E2F factors from wheat endosperm using the WDV element as bait (data not shown). A screen with the E2F *cis*-element from the promoter of the ribonucleotide reductase gene (RNR1b) from tobacco was more successful. The strong interaction of the human E2F5 protein with this element was demonstrated *in vitro *[26]. The fact that the first 94 quickly growing colonies selected in our screen with a triple repeat of the tobacco *cis*-element contained the full length sequence of the cDNA encoding the same *Ta*E2Fe factor reflects the very strong interaction of this factor with the bait. Unfortunately, it is not clear yet if other wheat E2F factors are unable to recognise this sequence or simply are not represented in the cDNA library or in the liquid endosperm fraction from which the library was prepared.

### False positives

Traditionally, Y2H screens are known to give a large amount of false positives [50,51]. The method of preparation of cDNA libraries and screening strategy for Y2H proposed by Clontech™ has several important improvements in comparison with other strategies. These eliminate some groups of false positives and sometimes give unambiguous results, but there is still no complete solution to the problem. Surprisingly, the Y1H screens of the same expressional cDNA libraries using known *cis*-elements give fewer false positives. In the case of strong protein-DNA interactions or the relatively high abundance of the cDNAs in the library (for example *Ta*E2Fe and *Ta*HD-ZipI TFs), most of the largest colonies were white in the α-galactosidase assay and encoded the expected type of transcription factors.

The blue colonies, which start to grow 3 to 4 days after appearance of quickly growing colonies, contain abundant cDNAs encoding some ribosomal proteins, histones, abundant housekeeping enzymes, *etc*. Some of these proteins appear to have the ability for non-specific interaction with a minimal promoter of all reporter genes; others (usually the latest colonies) are the result of the "leaky" growth on the -His medium because of non-optimal selection of the 3-AT concentration.

A further type of false positive specific for wheat grain cDNA libraries is a Zn-finger transcription factor which appears as the most abundant clone in the first portion of white colonies when specific protein-DNA interactions are weak or abundance of the cDNAs of interest is very low in the cDNA library (such as the screen of the cDNA library prepared from unstressed material with DRE *cis*-element).

This factor is a homologue of *At*VOZ1 and *At*VOZ 2 proteins from *Arabidopsis*, which specifically interact with a 38 bp region of the promoter of the vacuolar H^+^-pyrophosphatase (V-PPase) gene [52]. *At*VOZ2 binds as a dimer to the specific palindromic sequence, GCGTNx7ACGC. At least a few colonies containing the clone of the same wheat *At*VOZ-like protein were found in screens with several different reporter strains (data not shown). This result, and the finding that the expression of wheat *At*VOZ-like protein does not support growth of the non-modified Y187 strain on selective media, led us to conclude that part of the vector sequence that introduced into the yeast genome together with the bait sequence is recognised by this factor. As soon as the size and restriction pattern of the insert which encodes *At*VOZ-like protein is known, clones encoding this protein can be easily eliminated before plasmid purification and sequencing.

## Conclusion

In this paper an efficient way to use MATCHMAKER cDNA libraries for the yeast one-hybrid screen is proposed and demonstrated by cloning full length cDNAs of seven wheat transcription factors from several different families. Good correlation was found between abundance of particular clones in the cDNA library and the abundance of respective mRNAs in the RNA isolated from the liquid endosperm. The advantages of the method and possible false positives are discussed.

## Methods

### Generation of yeast reporter strains

Y1H reporter constructs were prepared by the cloning of tandems containing 3 to 4 repeats of the *cis*-elements for HDZipI-II, DREB/CBF, and E2F transcription factors into the pINT1-HIS3NB vector (see Table 1 for the DNA oligonucleotides used for *cis*-element preparation). The oligonucleotides with partially complementary sequences were annealed using Annealing Buffer (10 mM Tris-HCl, pH 7.5, 100 mM NaCl, 1 mM EDTA) and the resulting overhangs were used for cloning into the *Spe*I-*Not*I sites of the pINT1-HIS3NB vector. pINT1-HIS3NB vector (unpublished, kindly provided by Dr. PBF Ouwerkerk) was generated by cloning the *Not*I -*Bam*HI fragment of the pHIS3NB vector into the *Not*I-*Bcl*I unique sites of pINT1 [12]. Two unique restriction sites, *Not*I and *Spe*I, can be used for single step directional cloning of DNA fragments of interest into the binary vector. Constructs with inserts were selected by PCR using primers derived from the vector primers pINTseq and pINTseqr (Table 1) and sequenced using the same primers. Before yeast transformation, a fragment lacking the pUC29 sequence was isolated after restriction with *Nco*I-*Sac*I. Integration of the construct into the *PDC6 *locus of yeast genomic DNA *via *double crossover was performed as described in [12]. Reporter strains, designated yHDZipI-II, yDRE, and yE2F, were incubated overnight in the rich YPDA media, mixed with glycerol to 25% final concentration, and stored as 1 ml aliquots at -80°C until use.

### cDNA library construction

Bread wheat (*Triticum aestivum *L. cv. Chinese Spring) plants were grown in glasshouses with day temperatures of 18–25°C and night temperatures of 18–21°C, with a 13 h photoperiod. Spikes were labelled at anthesis. Pooled samples of liquid endosperm were collected at 3 to 7 DAP by pipette aspiration from the embryo sacs of dissected micropylar tips of the grain. Total RNA was isolated from wheat samples using TRI REAGENT (Molecular Research Centre, Inc., Cincinnati, OH), and 2 μg total RNA was used for the preparation of MATCHMAKER Two-Hybrid System 3 (Clontech, Palo Alta, CA) cDNA libraries according to the supplied manual, with the following modification: CHROMA SPIN+TE-1000 columns were used for the cDNA size fractionation, instead of the suggested CHROMA SPIN+TE-400 columns. The cDNA library was washed out from 100 plates with 300 ml YPDA containing 25% glycerol and 25 μg/ml kanamycin, and incubated for 30 min at 30°C with rotation (220 rpm). 1.5 ml aliquots of the cDNA library were frozen on dry ice and stored at -80°C until use.

### cDNA library screen

The conditions used for the one-hybrid screen are described in [12], with the following modification: instead of transformation of the reporter strain with plasmids prepared from an amplified cDNA library, we used overnight yeast mating with the AH109 strain, containing the library, prepared according to the protocol for the MATCHMAKER Two-Hybrid System Kit (Clontech™). 50 ml of overnight culture (alternatively, 1 ml of glycerol culture, thawed in a water bath at room temperature and diluted to 50 ml with YPDA) of each reporter strain were centrifuged for 5 min at 2000 rpm (Rotanta 460R table centrifuge, Hettich). The pellet was resuspended in 50 ml of 2 × YPDA medium containing 25 μg/ml kanamycin, mixed with a 1.5 ml aliquot of pre-made cDNA library in the AH109 yeast strain and incubated in a 2 l conical flask at 30°C overnight with slow (30–50 rpm) rotation. After 17–24 h of mating, the yeast cells were harvested, washed with 1 × TE buffer, pH 8.0, containing 25 μg/ml kanamycin, resuspended in 5 ml of the same buffer and spread on plates with selective media (-Leu, -His) containing 5–10 mM 3-amino-1,2,4-triazole (3-AT) to reduce possible leaky expression of HIS3 gene. The optimal concentration of 3-AT was determined before the screen as described in [12]. Library plasmids were isolated from positive colonies and analysed as described [9,12], except that the use of the α-galactosidase reporter gene (*MEL1*) allowed the selection of false positives (blue colonies) to be carried out directly on plates with the substrate X-α-GAL (25 μg/ml, Clontech™), as opposed to the filter assay required for strains with β-galactosidase gene as a reporter.

### Further characterisation of positive clones

Standard techniques were used for the manipulation of yeast cells (Yeast Protocols Handbook, PT3024-1, Clontech™ and as described elsewhere). Clones were grouped based on the length of PCR fragments obtained with T7 and 3' AD sequencing primers (pGADT7 vector information, PT3249-5, Clontech™) and restriction analysis of fragments with the same length. Yeast DNA of selected representatives of subgroups was transformed by electroporation into the strain DH5α of *E. coli*, plasmids were amplified and purified, and inserts were sequenced using T7 and 3'AD primers (pGADT7-Rec vector information, PT3530-5, Clontech™). In some cases, protein-DNA interactions were confirmed by retransformation of the reporter and control strains.

Multiple alignments of related protein sequences and the resulting dendograms (Fig. 4) were calculated using AlignX (Vector NTI Suite; Invitrogen, VIC, Australia). The Clustal W algorithm was used, with a gap opening penalty of 10 and a gap extension penalty of 0.05.

### Real Time PCR

Q-PCR was carried out according to [53] using the primer combinations shown in Table 2. The raw expression of the genes of interest was normalised with respect to the expression of a combination of three control genes, Glyceraldehyde-3-Phosphate Dehydrogenase (GAPdH), Cyclophilin (CyP) and Elongation factor alpha (eF-1 alpha) after the manner of [54].

**Table 2:** Q-PCR experimental details. A list of primer sequences, PCR product sizes and optimal acquisition temperatures for control genes and genes of interest used for the production and normalization of Q-PCR data.

Gene	Forward Primer	Reverse Primer	**PCR product size (bp)**	**Acquisition** ** Temperature (°C)**
*Ta*GAPdH	TTCAACATCATTCCAAGCAGCA	CGTAACCCAAAATGCCCTTG	220	79
*Ta*CyP	CAAGCCGCTGCACTACAAGG	AGGGGACGGTGCAGATGAA	227	83
*Ta*eF-1 alpha	CAGATTGGCAACGGCTACG	CGGACAGCAAAACGACCAAG	227	80
*Ta *HDZipI-1	CCAGATGGAGCACCACCACG	CACGCTCATTGCTGACCATG	198	83
*Ta *HDZipI-2	AGAGAGCGAGAGACTCGGTG	AAGAGCAGGGGCGGAAATGG	200	78
*Ta *HDZipII -1	AGACACCGGAGCAACGTACG	ACGAAGGACTAAACTTTCTG	180	83
*Ta *DREB2	GCGTACAACACCTTGATTTCC	AAACTCAACTCACATCTAAGC	182	75

### Availability of materials

The pINT1-HIS3NB vector will be made available for non-commercial purposes on written request to Dr PDF Ouwerkerk (E-mail: ouwerkerk@rulbim.leidenuniv.nl or p.b.f.ouwerkerk.2@umail.leidenuniv.nl). Other materials needed for cDNA library preparation can be purchased from Clontech (Palo Alta, CA).

## List of abbreviations used

AD – activation domain of GAL4

BD – binding domain of GAL4

CyP – Cyclophilin

DAP – days after pollination

eF-1 alpha – elongation factor alpha

GAPdH – glyceraldehyde-3-phosphate dehydrogenase

Q-PCR – quantitative (real-time) PCR

RT-PCR – reverse transcription – PCR

SD – selective media

TE – Tris-EDTA buffer, pH 7.5

UAS – upstream activator sequence

Y1H – yeast one-hybrid

Y2H – yeast two-hybrid

3-AT – 3-amino-1,2,4-triazole

## Competing interests

The author(s) declare that they have no competing interests.

## Authors' contributions

SL prepared the cDNA libraries and bait strains, adapted the Y1H method to screen yeast expressional cDNA libraries, prepared the yHDZip strain, screened for HDZip proteins, coordinated the work, and prepared the manuscript. NB prepared yDRE and yE2F bait strains, screened for E2F factors and performed all technical work like media preparation, plasmid isolation, sequencing reactions, *etc*. SM isolated DREB/CBF and MYB factors using the Y1H screen during her Honours project. ASM prepared the RNA tissue series for Q-PCR experiments and contributed to the manuscript. NS generated all Q-PCR data and worked on the manuscript.

PL, the author of the project, coordinated the work and contributed to the manuscript. All authors have read and approved the final manuscript.
